# Cross‐Sectional Area Measurement Techniques of Soft Tissue: A Literature Review

**DOI:** 10.1111/os.12757

**Published:** 2020-09-15

**Authors:** Xiao‐jing Ge, Lei Zhang, Gang Xiang, Yong‐cheng Hu, Deng‐xing Lun

**Affiliations:** ^1^ Beijing Wonderful Biomaterials Co., Ltd. Beijing China; ^2^ Beijing Ceramic Biotechnology Beijing China; ^3^ Weifang People's Hospital Weifang China

**Keywords:** Area measurement, Cross‐sectional area, Biomechanics, Soft tissues

## Abstract

Evaluation of the biomechanical properties of soft tissues by measuring the stress–strain relationships has been the focus of numerous investigations. The accuracy of stress depends, in part, upon the determination of the cross‐sectional area (CSA). However, the complex geometry and pliability of soft tissues, especially ligaments and tendons, make it difficult to obtain accurate CSA, and the development of CSA measurement methods of soft tissues continues. Early attempts to determine the CSA of soft tissues include gravimetric method, geometric approximation technique, area micrometer method, and microtomy technique. Since 1990, a series of new methods have emerged, including medical imaging techniques (e.g. magnetic resonance imaging (MRI), computed tomography (CT), and ultrasound imaging (USI)), laser techniques (e.g. the laser micrometer method, the linear laser scanner (LLS) technique, and the laser reflection system (LRS) method), molding techniques, and three‐dimensional (3D) scanning techniques.

## Introduction

The accurate assessment of the cross‐sectional area (CSA) of soft tissue (including tendons and ligaments) is a crucial prerequisite factor for estimation of biomechanical properties, such as Young's modulus, stress, elastic modulus, and energy density^1^, ^2^. Many techniques to measure the CSA of biological samples have been presented in the literature.

Historically, the methods for CSA measurement included the gravimetric method, the geometric approximation technique, the area micrometer method, and the microtomy technique. The gravimetric method, which calculates the CSA by dividing the specimen's volume (obtained from its weight) by the length, was frequently used in the 1960s. Some authors used dried specimens^3^, while others used wet specimens^4^, ^5^, ^6^. The geometric approximation technique assumes that the CSA of the tendon is rectangular^7^, ^8^, ^9^, ^10^, ^11^, ^12^, ^13^, round^14^, ^15^, ^16^, or elliptical^17^, ^18^, ^19^, ^20^, ^21^, ^22^, and measures the dimensions of the tendons by a micrometer or a microscope with a calibrated eyepiece micrometer. However, these methods, while convenient and non‐destructive, may introduce inaccuracies and are not suitable for soft tissue with non‐uniform shape. The area micrometer method allows CSA measurement of soft tissues with non‐uniform shape by compressing specimens into a slot of known width and then measuring the heights of the sections^23^, ^24^, ^25^. However, it has been proved to cause permanent damage to tendons^26^. When compared with more accurate methods, both the geometric approximation technique and the area micrometer method underestimate the CSA of most ligaments by approximately 15%–40%^27^. The microtomy technique can obtain shape information as well as the CSA by cutting fresh specimens^28^, ^29^, ^30^ or frozen specimens^31^, ^32^, ^33^ into sections and then projecting or digitalizing the cross‐sections. However, it is destructive and does not allow for subsequent biomechanical testing. Ellis compared seven CSA measurement methods for tendons^24^ and found that the gravimetric method obtained the best repeatability for dried specimens, and that the gravimetric method and the area micrometer method were comparable in terms of repeatability for fresh specimens.

Since 1990, a series of non‐destructive methods have emerged, including medical imaging techniques, laser techniques (e.g. the laser micrometer method, the linear laser scanner technique, and the laser reflection system), molding techniques, and three‐dimensional (3D) scanning techniques. Some researchers have attempted to measure soft tissue CSA using medical imaging techniques, such as magnetic resonance imaging (MRI)^34^, computed tomography (CT), and ultrasound imaging (USI)^35^. However, they faced problems of precision in small areas. Molding techniques determine the CSA of soft tissues by making casts of soft tissues using liquid‐silicon/polymethylmethacrylate (PMMA)^26^, ^27^ or alginate^36^, ^37^ and measuring their cast directly. Laser techniques use a laser beam to access the width or radius information and then reconstruct the cross‐sectional shape to access the CSA. Although the laser micrometer method is non‐destructive and accurate (<2%), and can obtain the relevant geometry information, it is unable to detect concavities and requires all‐round visibility^38^, ^39^. The laser reflection system (LRS) can detect cavities and is the first device that could measure changing CSA during tensile testing, but the strain rate must remain slow (approximately 2 mm/min)^40^. 3D scanning techniques access the CSA by sectioning the 3D model of soft tissues acquired by optical, laser, or ultrasound techniques, such as 3D ScanTop, structured light scanning (SLS), or 3D freehand ultrasound. Molding, laser, and 3D scan techniques can obtain the geometric information of soft tissues.

This review summarizes the principles and reliability of CSA techniques and indicates where they are most applicable. Five databases were searched (including PubMed, Embase, Cnki, Wanfang, and Vip databases) using an agreed set of keywords. Studies investigating the CSA techniques of soft tissue were eligible. After filtering by two authors, 119 studies were included. The comparison of all CSA measurement techniques is shown in Table 1. Information on the included studies is presented in Table 2.

**Table 1:** Comparison of cross‐sectional area (CSA) measurement techniques

Techniques	Dry/wet specimens	*In vivo*/*in vitro*	Destructive	Mechanism	Advantages and disadvantages
Gravimetric method	Dry/wet	*In vitro*	No	Divide the specimen's volume by the length. Volume can be determined by weight and density, specific gravity, or liquid displacement.	Convenient, repeatable. Unreliable and inaccurate compared with more recent techniques.
Geometric approximation technique	Dry/wet	*In vitro*	No	Assume the CSA of soft tissues was rectangular, round or elliptical and measures the dimensions of tendon.	Repeatable, convenient, and quick. May introduce non‐negligible inaccuracies and cannot detect concaves. Micrometers or Calipers may compress specimens to cause overestimations.
Area micrometer	Wet	*In vitro*	Yes	Squash soft tissues into a channel of known section until completely filled up and then measuring the heights of the sections using a micrometer.	Allow CSA measurement of soft tissue with non‐uniform shape, reproducible, and easy to handle. The instrument is relatively affordable, portable, and already commercialized. However, it is unable to obtain shape information, cannot be applied to dried specimens, will cause permanent damage to tissues, and will cause overestimation.
Microtomy technique	Dry/wet	*In vitro*	Yes	Microtomy technique determines CSA by staining and sectioning specimens for digitization. Some researchers sectioned fresh specimens, while others sectioned flash‐frozen specimens which is called the freeze‐fracture (cryomicrotomy) technique.	Allow morphometric measurements and suitable for almost all cross‐sectional geometry. However, it is destructive, and residual fat and bisection process may cause overestimation. Methyl blue penetration and crystallized water evaporation will reduce contrast.
Magnetic resonance imaging (MRI)	Wet	*In vivo*/*in vitro*	No	Exploit the spin density information in the sample to image.	Great contrast between tissues and image plane orientation can be set accurately and with 3D sequences. Allow multiple measurements. Can obtain morphological information, However, the relationship between the contrast in the MRI image corresponds with the actual borders of the tendon remains unknown and the equipment is expensive.
Ultrasonography	Wet	*In vivo*/*in vitro*	No	Emit and receive reflected ultrasonic waves and processing the signal to produce an image. Ultrasonography, in particular brightness mode (B‐mode) ultrasound, has been widely used.	It is readily available, relatively inexpensive, non‐destructive, and its temporal resolution is good, allowing for dynamic imaging and fast measurements. Portable devices are also available. Can obtain morphological information and allow repeated measurements. However, it needs expensive equipment and samples need to be immersed in a saline water bath, introducing the possibility of swelling. It needs all‐around visibility and reflection occurring at the boundary between two media may result in poor reliability to observe the borders.
Computed tomography (CT)	Wet	*In vivo*/*in vitro*	No	X‐ray radiological imaging technique which yields transverse tomographic images reflecting the spatial distribution of X‐ray attenuation in the part examined.	Easy, rapid, accurate, and commonly used diagnostic techniques. It can clearly define the boundary at the interface between fat and muscle. However, it is quite a complex and expensive technique not adaptable to all kinds of tests.
Molding techniques	Dry/wet	*In vitro*	No	Make a cast of soft tissues using liquid‐silicon or alginate and measure their cast directly.	Accurate, affordable, and relatively quick. However, this technique cannot be applied to measuring cross‐sectional area of more complex soft tissue (such as anterior cruciate ligament).
Laser micrometers	Dry/wet	*In vitro*	No	Reconstruct the cross‐sectional shape based on the width measurements obtained by collimated laser beams.	Non‐destructive, can obtain geometry information, and allow multiple measurement. However, the concave region is assumed to be flat. It needs all‐round visibility and the data acquisition process is relatively slow. The operations are complicated.
Laser reflection system (LRS)	Dry/wet	*In vitro*	No	Rotate laser sensor around the soft tissue samples in a circular path and then reconstruct the cross‐sectional shape and determine cross‐sectional area by the inner radius (r) of the specimen relative to the rotating center and corresponding angle in polar coordinates using Simpson's rule.	Can detect concavities in an accurate, repeatable, and rapid manner. It is a non‐contact, non‐destructive and accurate tool and can measure changing CSA during tensile testing. However, a complete revolution of the LRS takes over 20 s to complete and the strain rate must be slow (approximately 2 mm/min), which may introduce error caused by the viscoelastic of soft tissues. The rotation center should always be in the cross‐section of the specimen. It is not suitable for semitransparent surfaces. Only specimens larger than 20 mm^2^ were considered currently. A steep angle between laser beams and the target surface may produce artifact.
Linear laser scanner (LLS)	Dry/wet	*In vitro*	No	Two charge‐coupled device (CCD) laser reflectance devices mounted facing each other horizontally sweep the specimen and measure the distance from the laser to the tendon surface, followed by image reconstruction.	Accurate, noncontact, repeatable, and fast and easy to assemble and operate. It can adapt to various types of testing machines and is capable of moving during testing. Do not need precise centering and can perform during mechanical testing. However, this device cannot precisely acquire steep concavities due to the reflection of the laser beams and the specimen maximum measurable thickness (45 mm) is also a limitation.
3D‐scanning techniques	Dry/wet	*In vivo*/ *In vitro*	No	Access the cross‐sectional area by sectioning the 3D model of soft tissues acquired by optical, laser, or ultrasound techniques, such as structured light scanning (SLS) and 3D freehand ultrasound.	It is Accurate, repeatable, non‐contact; it allows multiple measurement and can obtain morphological information. However, it needs all‐around visibility and can only achieve accurate results during static conditions. The accuracy of results may not be representative of the accuracy of measurements *in vivo*. The cost may be a limitation.

**Table 2:** Information of included studies

First Author	Year	CSA measurement method		Samples	Comments
Geometric method					
Haut ^17^	1969	Geometric	Elliptical	Canine ACL	
Boyer ^18^	2001	Geometric	Elliptical	Flexor digitorum profundus and flexor pollicis longus tendons	
Erhard ^19^	2002	Geometric	Elliptical	Flexor digitorum profundus tendons	
Joyce ^20^	2014	Geometric	Elliptical	Porcine flexor tendons	
Yilmaz ^21^	2016	Geometric	Elliptical	ACL and PCL	
Yang ^22^	2018	Geometric	Elliptical	Achilles tendon	Diameter is measured by ruler and vernier caliper.
Woo ^8^	1980	Geometric	Rectangular	Porcine medial and lateral digital extensors	Designed a precision apparatus used to measure the thickness of tendon.
Hanson ^9^	1998	Geometric	Rectangular	Lumbar anterior longitudinal ligament	
Matsumoto ^10^	2003	Geometric	Rectangular	Rabbit Achilles tendon	Digital micrometer
Huang ^11^	2004	Geometric	Rectangular	Rat Achilles tendon	
Langenderfer ^12^	2006	Geometric	Rectangular	Tendons	
Johnson ^41^	2008	Geometric	Rectangular	Foot ligament	
Legerlotz ^15^	2010	Geometric	Round	Tendon fascicles	
Cavaignac ^16^	2014	Geometric	Round	Hamstring, gracilis tendon, semitendinosus tendon, ACL	The diameter was measured by tying a suture around the graftand placing a 0.5 N load on it
Gravimetric approximation technique					
Infantolino ^42^	2010	Gravimetric	Density	First dorsal interosseous tendon	Moist specimens, CSA = Mt./(Lt*ρ) Mt‐Weight of tendon; Lt‐Length of tendon; ρ‐density of tendon 1120 kg*m̂(−3)
O'Brien ^43^	2010	Gravimetric	Volume	Quadriceps muscle	Muscle volume was measured by the serial axial‐plane MRI. CSA = muscle volume/optimum fascicle length
Reece ^44^	2011	Gravimetric	Density	Upper and lower limb tendon	
Area micrometer (AreaM)					
Butler ^25^	1986	AreaM		ACL, PCL, Patellar tendon, the lateral collateral ligament	Accurate to 0.01 mm^2^
DeBerardino ^45^	2008	AreaM		Achilles tendon	
Donahue ^46^	2001	AreaM		Bovine digital extensor and human hamstring tendons	Ellis's area micrometer.
Ellis ^24^	1969	AreaM		Cat extensor digitorum communis tendons	
Jackson ^47^	1993	AreaM		Goat patellar tendon	0.12 MPa for 2 min.
Nagasaki ^48^	2006	AreaM		PCL, meniscofemoral ligaments	0.12 MPa for 2 min. Area micrometer is from Mitutoyo and Jasco, Japan.
Race ^26^	1996	AreaM		Semitendinosus tendon	Using Butler's area micrometer and displacement was measured by an LVDT. The mean difference between it with molding technique was 16.2 ± 6.9% (SD).
Toritsuka ^49^	2003	AreaM	Oval‐shaped	Bone‐Patellar Tendon‐Bone, hamstring tendon	0.12 MPa
Walker ^23^	1964	AreaM		Plantaris tendon	Three instruments with slots of 0.066, 0.163, and 0.248 in. in width.
Microtomy technique					
Cronkite ^28^	1936	Microtomy	Fresh	Tendons	0.5–1 mm slices. The outline of the tendon was traced with pencil andthe area measured with a planimeter.
Itoi ^29^	1995	Microtomy	Fresh	Shoulder tendons	Distal, middle, and proximal slices. Using an image analysis system (JAVA; Jandel video analysis system, Jandel Scientific, Corte Madera, CA).
Yoganandan ^50^	2000	Microtomy	Fresh	Cervical spine ligaments	Particular cross‐section was projected on a paper and an outline of the ligament boundary was traced and analyzed using a computer‐aided design (CAD) program.
Strauss ^30^	2007	Microtomy	Fresh	Adductor longus tendon	SigmaScan Pro Image Analysis software
Iriuchishima ^51^	2014	Microtomy	Fresh	ACL	The outline of ACL was marked with colored ink. Image J. The CSA of HT and BPTB is assumed to be round and rectangular.
Pintar ^31^	1992	Microtomy	Frozen	Lumbar spine ligaments	1 mm slices. Particular cross‐section was projected on a paper and an outline of the ligament boundary was traced and analyzed using a computer‐aided design (CAD) program.
Buchanan ^32^	2001	Microtomy	Frozen	Chicken Achilles tendon	Tendons were embedded in Tissue‐Tek OCT compound and quick frozen in liquid nitrogen. CSA was measured using NIH Image.
Mkandawire ^33^	2005	Microtomy	Frozen	Foot and ankle ligaments	Compared with the freeze‐fracture technique, the digital caliper method resulted in an approximately 35% difference in cross‐sectional area.
Ahmad ^52^	2007	Microtomy	Frozen	Biceps tendon	
Conrad ^53^	2013	Microtomy	Frozen	Achilles tendons	2 mm tendon sections. CSA was measured using Image J.
Zens ^54^	2015	Microtomy	Frozen	Anterolateral ligament	Quick‐frozen in liquid isopentane. Five sections.
Magnetic resonance imaging (MRI)					
Couppe ^55^	2014	MRI	1.5 and 3 T	Equine Patellar tendon	Compared with mold casting technique, MRI underestimated CSA for approximately 2.8%.
Fujimaki ^56^	2016	MRI	3 T	Cadaveric ACL	Evaluate the accuracy of MRI for the noninvasive clinical assessment of CSA and compared with laser‐scanner indicating that the MRI method was highly repeatable and reliable for estimating the CSA of the ACL
Garau ^57^	2018	MRI	3 Tfast spin‐echo	Extraocular muscle	
Bickel ^58^	2008	MRI	1.5 T	Hamstring tendon	Preoperative determination of graft size
Grawe ^59^	2016	MRI	1.5 T and 3 T	Hamstring tendon	Modified the technique described by Bickel
Hallgren ^60^	2019	MRI	3 T	Rectus capitis posterior minor	
Hanna ^41^	2019	MRI	1.5 T and 3 T	Hamstring tendon	MRI CSA measurement of the average STGR (semitendinosus tendon CSA added to the GR CSA) was a significant predictor of graft size
Murray ^61^	2004	MRI	1.5 T	Equine DDFT	
Schramme ^62^	2010	MRI	1.5 T	Equine SDFT	
Stenroth ^63^	2019	MRI	0.18 T	Achilles tendon, Patellar tendon	Compared with ultrasound imaging, MRI provides superior reliability for tendon CSA measurements. Tendon CSA was imaged from three locations to estimate average tendon CSA and segmented using OsiriX software.
Wernecke ^64^	2011	MRI	1.5 T	Gracilis tendon, Semitendinosus tendon	Preoperative MRI is a clinically useful tool to assess hamstring graft diameter.
Ultrasound imaging technique (USI)					
Richards ^65^	2001	USI	Power doppler ultrasound	Achilles tendon	
Noguchi ^35^	2002	USI	Tracing software	Rabbit Achilles tendon, Medial collateral ligament, ACL	A method of *in vitro* measurement of the cross‐sectional area of soft tissues, using ultrasonography. There is no significant difference between the use of ultrasonography and digital calipers, which implies that ultrasonography would also underestimate the cross‐sectional area.
Muraoka ^66^	2004	USI	B‐mode	Achilles tendon	
Toprak ^67^	2012	USI	Tracing software	Patellar tendon	
Gellhorn ^68^	2013	USI	Tracing software	Patellar tendon	Inter‐rater, intra‐rater, and inter‐machine reliability of quantitative ultrasound measurements. Bland–Altman plots demonstrated a mean difference between sonographers of 0.03 mm̂2 for CSA measurements.
Bohm ^69^	2016	USI	Tracing software	Achilles tendon	The comparison of the ultrasound‐based and MRI‐based tendon CSA determination showed a significant main effect (po0.05) of the factor method. Ultrasound‐based methodology cannot be recommended for an accurate Achilles tendon CSA determination *in vivo*.
Galanis ^70^	2016	USI	Tracing software	Semitendinosus tendon Gracilis tendon	Compared with 1.5 T MRI
Bisi‐Balogun ^71^	2017	USI	Tracing software	Plantar fascia	Using this novel technique, PF CSA, and width may be determined reliably using measurements from one sonogram or the mean of three sonograms.
Kojah ^72^	2017	USI	Tracing software	Equine SDFT, DDFT	Transverse ultrasound images to measure the CSA can be stated as a technique with high precision and accuracy.
Martin ^73^	2018	USI	Elliptical	Achilles tendon	
Naghibi ^74^	2019	USI	3 T	Cadaver Knee ligaments	High‐frequency ultrasound system
Computed tomography (CT)					
Haggmark ^75^	1978	CT		Thigh muscle	The CSA of the thigh muscle can be accurately determined by CT.
Durrington ^76^	1982	CT		Achilles tendons	The total number of pixels in the tendon to determine CSA.
Sipila ^77^	1993	CT		Quadriceps muscle	Compared CT with USI to measure muscle cross‐sectional areas. The CSA measured by USI correlated highly with CT.
Strandberg ^78^	2010	CT		Thigh muscle	ImageJ as a method to evaluate CSA. The method shows an overall excellent reliability with respect to both observer and replicate.
Sherk ^79^	2011	CT	pQCT (strong filter)	Midthigh muscle	CSAs did not differ significantly between MRI and strongly filtered pQCT images (within 4%). A strong filter on pQCT images can reduce noise.
Ozola‐Zalite ^80^	2019	CT		Skeletal muscle	Compare the reliability and validity of a newly developed segmentation software VikingSlice against SliceOMatic for quantification of adipose tissue and skeletal muscle cross‐sectional areas (CSA)
Molding technique					
Race ^26^	1996	Molding	Silicone rubber	PCL	Invented a new replica molding technique for CSA measurement and investigated the accuracy by comparing with area micrometer and laser micrometer. Relative to the corrected replica method, it was calculated that the area micrometer underestimated the cross‐sectional area by 16.2 ± 6.9% (SD) and that the assumption of a convex cross‐section would have caused the laser micrometer to overestimate cross‐sectional area by an average of 2.3 ± 1.5% (SD) for tendon. The CSA was determined by square counting
Muneta ^81^	1997	Molding	Silicone rubber	ACL	Silicone rubber (GC Fujirock, GC, Tokyo, Japan), analyses by Shadow Graph model 6.
Gupte ^82^	2002	Molding	Silicone rubber	Meniscofemoral ligaments	Race–Amis casting method. The CSA were measured using Scion Image software.
Schmidt ^27^	2010	Molding	Silicone rubber	Foot ligaments	Materials were optimized so that the mold could be used for multiple castings and so the castings would not shrink. CSA was determined by Image analysis software.
Goodship ^36^	2005	Molding	Alginate	Equine SDFT	This technique is quick and simple to carry out and provides accurate values (within 0.8%) for CSA which are reproducible (coefficient of variation = 1.42%)
Stieven Filho ^83^	2015	Molding	Alginate	Bovine extensor digitorum tendons	Using Type II Jeltrate alginate and Image‐Pro Plus software
Colaco ^37^	2017	Molding	Alginate	Bovine tendons	Goodship's method. Blueprint Cremix alginate and Image J.
Zaino ^84^	2017	Molding	Alginate	Deer ACL	Using Jeltrate Plus alginate and Image J.
Bauer ^85^	2018	Molding	Alginate	Porcine tendons	Using Type II Jeltrate alginate and Image‐Pro Plus software
Ahmadzadeh ^86^	2019	Molding	Alginate	Patellar tendon	Goodship's method.
Laser technique					
Lee ^38^	1988	Laser	Laser telemetric system	Porcine ACL	The maximum error in CSA is less than 4%. Cannot detect concavities in a specimen. Procedure is slow.
Woo ^39^	1990	Laser	Laser micrometer	Rabbit medial collateral ligament, ACL	The areas obtained by the laser micrometer system were compared with digital calipers and area micrometer. Digital calipers and constant pressure area micrometer were 16–20 percent lower.
Carlson ^87^	1993	Laser	Laser micrometer	Palmaris longus, plantaris and extensor digitorum longus tendons	Sequential measurements demonstrated the cross‐sectional shape along the longitudinal axis of the tendon.
McGough ^88^	1996	Laser	Laser micrometer	Biceps tendon	Treat tendons into dumbbell shape.
Harner ^89^	1999	Laser	Laser micrometer	PCL	The sample is perpendicular to the ground during the measurement
Moon ^90^	2006	Laser	LRS	Goat Achilles tendon, porcine ACL	A laser reflectance system using a charge‐coupled device (CCD) laser displacement sensor was developed and tested.
Favata ^91^	2006	Laser	LRS	Rat patellar tendons	Improve CSA measurement method for small connective tissues using a laser triangulation sensor, which determines position by measuring reflected light from the target surface; the accuracy and repeatability of the system were demonstrated.
Pokhai ^40^	2009	Laser	LRS	Bovine tendons	It is capable of measuring changing CSA of soft tissues during tensile testing. CSA values measured by the LRS and the casting method were consistently lower than those obtained by molding sectioning.
Peltz ^92^	2009	Laser	LRS	Rat supraspinatus and multiple rotator cuff tendons	Favata's method.
Rooney ^93^	2014	Laser	LRS	Rat supraspinatus tendon	Peltz's method.
Weber ^94^	2015	Laser	LRS	Forearm tendons	Apply Pokhai's method on human tendon and it shows great accuracy.
Vergari ^95^	2010	Laser	Linear Laser Scanner	Equine SDFT	Design a noncontact, fast, and accurate device capable of acquiring CSA of specimens mounted on a testing machine. Measurements of the geometrical shapes yielded mean errors lower than 1.4%
Vergari ^96^	2011	Laser	Linear Laser Scanner	Equine SDFT	
Bruneau ^97^	2010	Laser	Optic micrometer	Rat Tail Tendons	With the profile reconstruction algorithm and optic micrometer, we are able to estimate the cross‐sectional area within a 2% margin of error.
Three‐dimensional (3D) scanning techniques					
Hashemi ^98^	2005	3D	3d ScanTop imaging system	ACL	A commercially available photographic scanner, 3‐D Scantop (Olympus America), was used to construct the 3‐D image of human ACL.
Barber ^99^	2009	3D	Free‐hand 3D Ultrasound (FUS)	Gastrocnemius medialis	Assess the validity and reliability of FUS and compare that with MRI. FUS could be used as an alternative to MRI for measuring *in vivo* muscle morphology and PCSA.
Benard ^100^	2011	3D	Free‐hand 3D Ultrasound (FUS)	Gastrocnemius medialis	
Obst ^101^	2014	3D	Free‐hand 3D Ultrasound (FUS)	Achilles tendon	Investigated the accuracy and the reliability of *in vivo* Achilles tendon (AT) CSA measurements obtained using freehand 3D ultrasound.
Hayes ^102^	2016	3D	Structured light scanning (SLS)	Rabbit Achilles tendon	Present a simple method, using structured light scanning (SLS), to acquire a 3D model of rabbit Achilles tendon *in vitro* for measuring CSA of a tendon.
Dickinson ^103^	2018	3D	CT	Monkey jaw‐adductor muscles	Diffusible iodine‐based contrast‐enhanced computed tomography (diceCT). It enables high‐resolution data.
Keuler ^104^	2019	3D	3 T scanner	Achilles tendon	Three‐dimensional volume reconstructions.

Note: Samples that are not identified are all from humans. Classified by CSA measurement technique and ordered by year.

ACL, anterior cruciate ligament; DDFT, deep digital flexor tendon; PCL, posterior cruciate ligament; SDFT, superficial digital flexor tendon.

## Methods

Five databases were searched, including PubMed, Embase, Cnki, Wanfang, and Vip databases. The strategy had three components: soft tissue, CSA, and measurement. Two independent reviewers assessed the potential studies retrieved by EndNote, with any disagreements mediated by a third reviewer. Once duplicates were removed, titles, abstracts, and full texts of the studies were screened for eligibility according to the following inclusion criteria: (i) studies that were published as full reports before December 2019; (ii) studies that investigated the CSA measurement techniques of soft tissues; and (iii) studies that investigated the CSA measurement of airways, nerves, and muscle fibers. Finally, 119 studies were included in this review.

## Gravimetric Method

The gravimetric method calculates the CSA of soft tissues by dividing the specimen's volumeby the length. Volume can be determined by weight and density, specific gravity, or liquid displacement.

The gravimetric method was frequently used in the 1960s for CSA measurement of moist specimens^4^, ^5^, ^6^ and dried specimens^3^. Ellis^24^ compared four gravimetric methods (including moist specimen weight per unit length, moist specimen displacement volume per unit length, dry specimen weight per unit length, and dry specimen displacement volume per unit length) with area micrometer, shadow amplitude contour reconstruction, and planimeter measurements on photomicrographs of histological sections. Results showed that dry specimen weight per unit length had the best repeatability on single measurements for CSA of tendon specimens, and moist specimen gravimetric measurement and the area micrometer method were significantly more repeatable than shadow amplitude contour reconstruction.

This method made a considerable contribution in the early days: it can provide an approximate CSA and it is repeatable. However, errors are inevitably introduced. In addition, it is unreliable to determine the volume of soft tissues by water displacement, density, and weight. Cronkite (1936) found that this method did not provide consistent results^28^.

## Geometric Approximation Technique

The geometric approximation technique assumes that the CSA of soft tissues are rectangular^7^, ^8^, ^9^, ^10^, ^11^, ^12^, ^13^, round^14^, ^15^, ^16^, or elliptical^17^, ^18^, ^19^, ^20^, ^21^, ^22^, and measures the dimensions of tendons. Dimensions can be measured by ruler, vernier caliper, digital micrometer, microcaliper^30^, or microscope with a calibrated eyepiece micrometer.

**Fig. 1:**
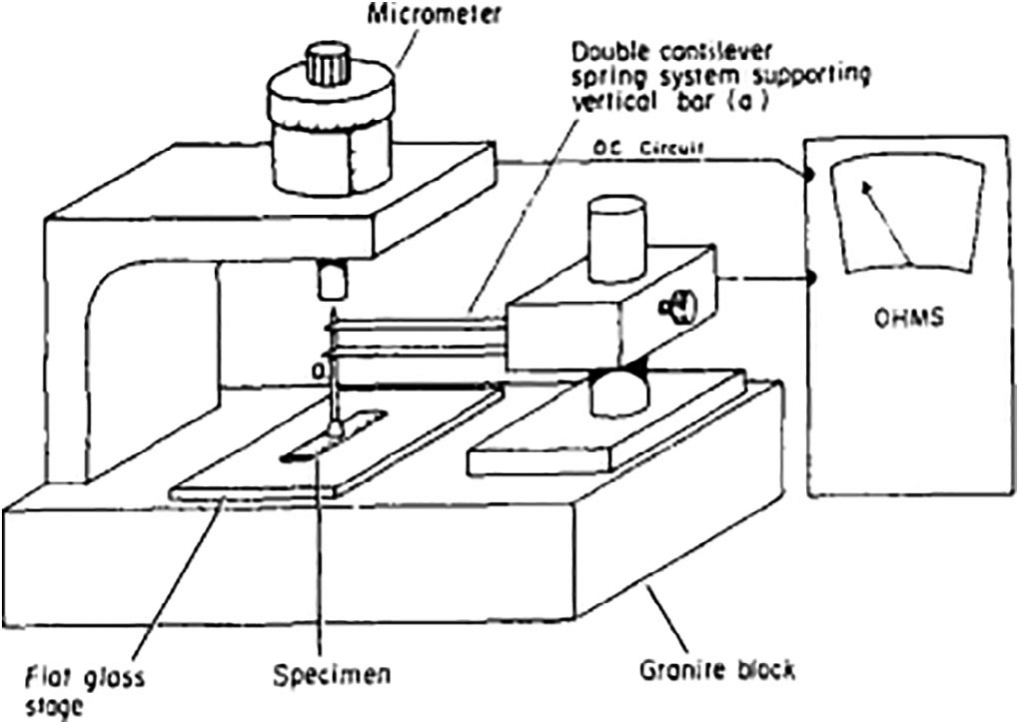
A precision apparatus used to measure the thickness of tendon designed by Woo *et.al*., in which a vertical rod supported by a double cantilever spring system was placed on the specimen. Then a minimal vertical force was applied on the tendon by the rod and the spindle of the micrometer was then lowered to contact the sharp point on the top of the vertical rod. An electrical circuit was used to indicate contact^8^.

Haut and Little assumed the cross‐sectional shape of the canine anterior cruciate ligament (ACL) to be elliptical and calculated the CSA by measuring the major and minor axes with a micrometer^17^. Woo et.al assumed the cross‐sectional shape to be rectangular and developed a micrometer instrument that applied a minimal compressive force to the specimen for thickness determination (Fig. 1), while the widths of specimens were measured with a cathetometer^8^. Matsumoto compared the CSA of rabbit Achilles’ tendons measured by micrometer with CSA measured by observing tendon sections under a microscope, and found that the results did not differ^10^.

The geometric approximation technique is repeatable, convenient, non‐destructive, and can be performed quickly. It can be applied for both fresh and dried tissues. However, it may introduce non‐negligible inaccuracies and is not suitable for soft tissue with complex and non‐uniform shape or concave surfaces. Micrometers or Calipers may compress specimens, resulting in overestimation.

## Area Micrometer Method

The area micrometer method determines the CSA of soft tissues by squashing them into a channel of known section until completely filled up and then measuring the heights of the sections by means of a micrometer head mounted upon the instrument.

**Fig. 2:**
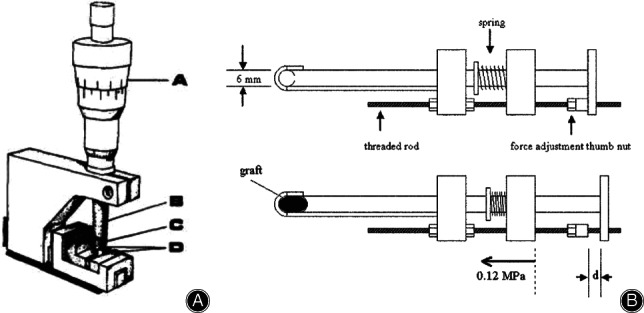
(A) Constant area micrometer designed by Ellis which consists of torque measuring thimble (A), spindle (B), plug (C), and side blocks (D). The specimen was placed between blocks (D) and beneath the blade portion of the plug (C). The thimble of the micrometer head was turned to bring the spindle into contact with the plug until the thimble torque reached its prescribed value. The thimble was then released and the micrometer read^24^. (B) Oval‐shaped slot area micrometer designed by Toritsuka. The graft was placed into an oval‐shaped slot 6 mm in width and a constant pressure of 0.12 MPa was applied with an attached spring. The sliding caliper was read and then the CSA was calculated^49^.

The area micrometer method was first developed by Walker *et al*. (1964) and was applied to measure the CSA of human tendons^23^. It consisted of a rectangular slotted plate with a movable following bar. Three instruments with different slot width are fabricated to permit measurements of tendons of a wide range of CSA. Results showed that the area micrometer had great reproducibility and the error was less than 1%. To better control the pressure to reduce errors caused by pressure, the constant pressure area micrometer was invented by Ellis *et al*.^24^, which could squash the tissue with a constant force and read CSA from a suitably calibrated linear variable differential transformer (LVDT) or dial gauge (shown in Fig. 2A). In the operation, the specimen was placed between a pair of stainless‐steel side blocks which were positioned 0.0307 inch apart. In 2003, an area micrometer with an oval‐shaped slot was customized^49^ (shown in Fig. 2B). Specimens are placed into the oval‐shaped slot and a constant pressure of 0.12 MPa is applied with an attached spring. The sliding caliper is read and then the CSA is calculated from this displacement using the following formula: CSA = 3 × 3 × 3.14 + 6 d.

Pressure is a key factor for the area micrometer. Sufficient pressure must be applied to prevent overestimation, but excessive pressure may cause damage to tissues. With the increase of press time, tissue specimens will be stiffer due to the loss of liquid/ground substance^26^. Standard values of 0.12 MPa applied pressure and 2 min deformation time are ideal^25^.

In conclusion, the area micrometer method allows the measurement of CSA of fresh soft tissue with non‐uniform shape. It has great reproducibility^24^ and is easy to use. The instrument is relatively affordable, portable, and already commercialized. Although there are many more accurate alternatives, the area micrometer method is still popular and widely used. However, it has several obvious shortcomings. This technique is also unable to obtain shape information and cannot be applied to dried. specimens Moreover, the area micrometer method consistently compresses specimens with pressure and confines them to a specific shape. Therefore, it has been proved to cause permanent damage to tendons.^26^ When compared with more accurate methods, both the geometric approximation technique and the area micrometer method overestimate the CSA of most ligaments by approximately 15%–40%^27^.

## Microtomy Technique

The microtomy technique determines CSA by staining and sectioning specimens for digitization. Some researchers section fresh specimens, while others section flash‐frozen specimens, which is called the freeze‐fracture (Cryomicrotomy) technique. Liquid nitrogen is usually used as the freezing agent. Soft tissue is coated with a methyl blue dye to enhance contrast during imaging.

**Fig. 3:**
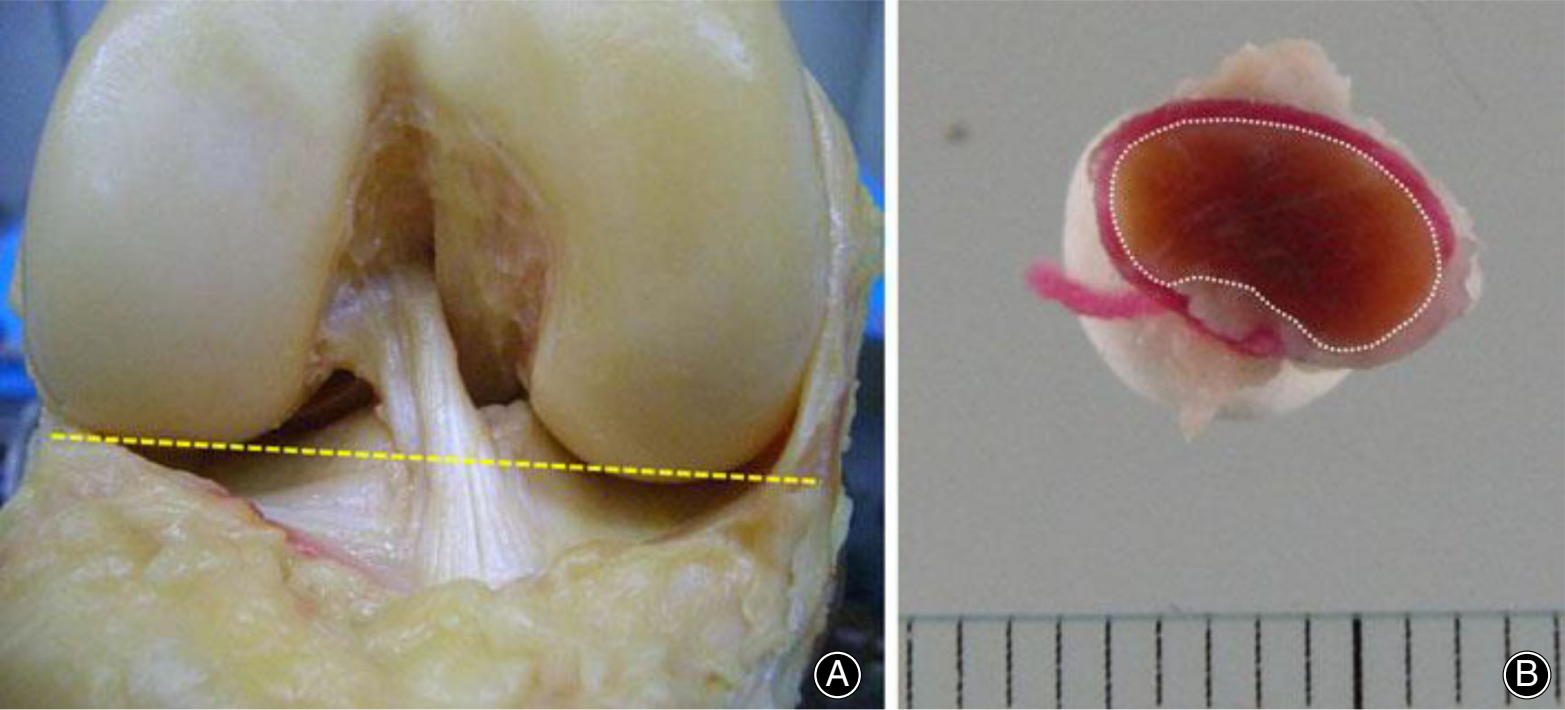
Application of microtomy technique on anterior cruciate ligament (ACL) cross‐sectional area measurement. (A) Slice plane of ACL cross‐section. (B) ACL cross‐sectional area measurement using Image J software. The surface of ACL is stained by colored ink for enhanced contrast. Ruler provided calibration data^51^.

In 1936, Cronkite presented a microtomy method that obtained the CSA of fresh tendons by cutting sections 0.5‐mm to 1.0‐mm thick from tendon specimens with a thin razor blade, projected shadows of them, and measured CSA with a planimeter^28^. He compared this technique with methods that obtain CSA by dividing volume with length and found the section method to be more accurate. In 2014, Iriuchishima measured the anterior cruciate ligament (ACL) mid‐substance CSA by sectioning fresh ACL, as shown in Fig. 3 ^51^. However, without freezing, it is hard to obtain uniform sections and to cut sections perpendicular to the long axis of specimens.

Mkandawire later developed a freeze‐fracture technique for measuring ligament CSA to determine human foot and ankle ligament morphometry, in which ligaments were quick‐frozen with an immersion technique using isopentane chilled by a liquid nitrogen bath^33^. Isopentane has great stability at subzero temperatures and is inert to human tissue. Then, ligaments were bisected by hammer and ground wood chisel. Methyl blue and liquid paper were used for enhancing contrast. The cryomicrotomy technique has been applied to obtain the geometric and mechanical properties of human cervical spine ligaments and lumbar spinal ligaments^31^, ^50^. In Buchanan's research, tendon specimens were embedded in Tissue‐Tek OCT compound and quick frozen in liquid nitrogen^32^.

On the one hand, the cryomicrotomy technique allows for morphometric measurements of soft tissues and is better adapted to measurement of complex cross‐sectional geometry, such as short ligaments (less than 10 mm) and ligaments with bony projections. On the other hand, the cryomicrotomy technique is destructive and does not allow for subsequent biomechanical testing and multiple measurements^33^. In addition, residual fat may cause overestimation. Methyl blue penetration and crystallized water vaporized during the immersion process will reduce contrast. Shearing generated during the ligament bisection may result in overestimation.

## Medical Imaging Techniques

Some researchers have attempted to measure soft tissue CSA using medical imaging techniques, such as MRI and ultrasound. All three medical imaging techniques can be used for measuring CSA *in vivo*.

### *Magnetic Resonance Imaging*

The MRI technique exploits the spin density information in the sample to image; therefore, it is completely non‐invasive^105^. It has long been used to qualify changes in muscle volume and CSA after exercise.

**Fig. 4:**
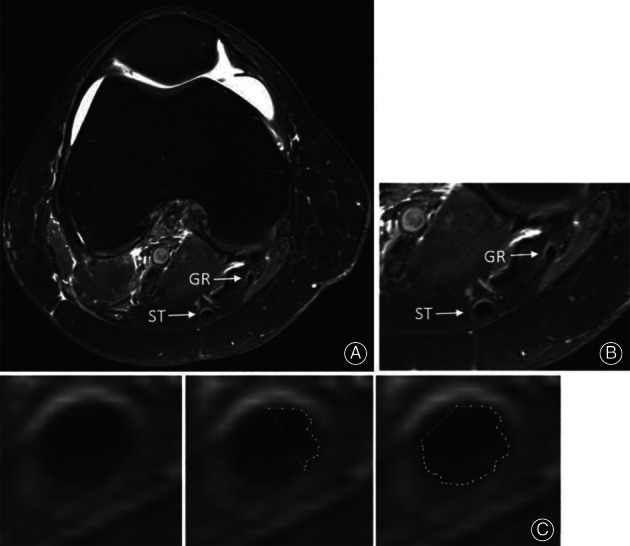
Magnetic resonance image (MRI) image for hamstring tendon cross‐sectional area (CSA) measurement. (A) Axial view of the right knee at the widest point of the medial femoral condyle. (B) Magnified view at the same level showing the semitendinosus (ST) and gracilis (GR) tendons. (C) Demonstration of the use of the region‐of‐interest tool to trace the cross‐sectional area of the ST tendon^41^.

In 2001, Anderson *et al*. used MRI technology to measure the CSA of the anterior cruciate ligament ACL^34^. However, they assumed the CSA of soft tissue was elliptical and MRI fails to capture the details of the boundary due to resolution limitations. In addition, rotation during imaging will change the shape and dimensions of soft tissues, so it would be difficult to make sure soft tissue always line up with the imaging plane and, thus, obtain an oblique section. Stenroth suggested that MRI provides superior reliability for tendon CSA measurements compared with USI^63^. MRI has been suggested to be the gold standard for measuring the tendon CSA to investigate the validity of USI‐based methods^69^, ^106^. However, Couppe noted that tendon CSA measured by MRI is associated with an underestimation compared with the molding technique, but by optimizing the measurement using a 3 Tesla MRI and the appropriate National Institutes of Health color scale, this underestimation could be reduced to 2.8%^55^. MRI CSA measurement can also be used to predict the expected ACL autograft size^41^. Figure 4 shows the MRI image for hamstring tendon CSA measurement, which traces the CSA using of the region‐of‐interest tool.

Magnetic resonance imaging provides great contrast between tissues and there is no pressure applied on the tissue. Image plane orientation can be set accurately, and with 3‐D sequences, the imaging plane can be adjusted post‐imaging (re‐slicing)^63^, ^107^. However, how well the contrast in the MRI image corresponds with the actual borders of the tendon remains unknown, and, as a result, the MRI‐based measurement may either underestimate or overestimate the tendon CSA.

### *Ultrasound Imaging*

Ultrasonic imaging involves emitting and receiving reflected ultrasonic waves and processing the signal to produce an image. The higher the frequency, the weaker the diffraction and the higher the image resolution. However, high frequency may also result in poor penetration. Ultrasonography, in particular brightness mode (B‐mode) ultrasound, has been widely used to quantify the CSA and to investigate the morphologic and mechanical properties of soft tissues *in vivo* and *in vitro* ^101^.

**Fig. 5:**
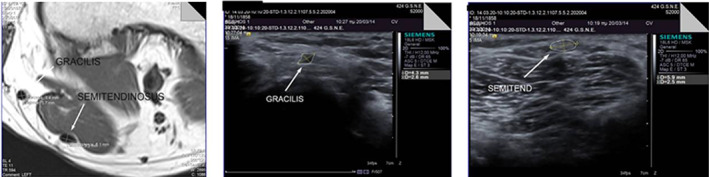
*In‐vivo* ultrasound imaging technique for cross‐sectional area (CSA) measurement of semitendinosus and gracilis tendon. Upper graph: axial T1‐weighted sequence of a left knee magnified four times. The semitendinosus tendon (ST) and gracilis tendon (GT) CSA are displayed. Lower panel: Ultrasound images and cross‐sectional area of the ST and gracilis tendon calculated with the ellipse tool (white dotted lines) of the ultrasound device^70^.

**Fig. 6:**
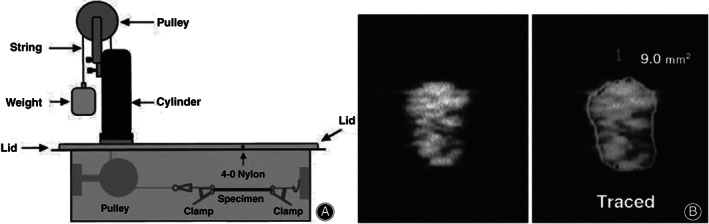
(A) Schematic drawings of a specially designed *in vitro* ultrasound cross‐sectional area (CSA) measurement device. The inside of the device is filled with a saline solution. Both ends of the specimen are held by clamps. A weight is then connected to the string to prevent the specimen from sagging. (B) Ultrasonogram of a specimen and its CSA, which is assumed to be the sum of the pixels in the enclosed area^35^.

Diagnosing tendinopathy often involves the measurement of tendon size using diagnostic USI. Brushoj *et al*. and Richards *et al*. investigated the reliability of MRI and diagnostic USI in CSA measurement of Achilles and tibialis tendons and results indicated high intra rater‐and inter‐rater reliability between USI and MRI^65^, ^108^. Galanis *et al*. found that the ICC among the USI, MRI, and intraoperative graft methods for the semitendinosus tendon and the GT data ranged from 0.502 to 0.906^70^. Figure 5 shows the USI of the semitendinosus and the gracilis tendon CSA measurement. In 2002, Noguchi *et al*. designed an *in vitro* ultrasonography‐based system to determine the CSA of the tendon and found that there is no significant difference between the use of ultrasonography and digital calipers, which implies that ultrasonography would also underestimate the CSA^35^, the schematic of which is shown in Fig. 6.

Ultrasound imaging is an appealing method of choice for scientific research and clinical evaluation. It is readily available, relatively inexpensive, and non‐destructive, and its temporal resolution is good, allowing for dynamic imaging and fast measurements. Portable devices are also available. This method can also obtain morphological information of the sample section and can be repeated at will without damage to the tissues. However, expensive equipment is required and samples need to be immersed in a saline water bath, introducing the possibility of swelling. It has also proved to be inaccurate^69^. Furthermore, bone needs to be resected to provide clear vision. Moreover, reflection occurs at the boundary between two media with different acoustic densities, which may result in poor reliability to observe the borders and, thus, cause overestimation or underestimation of CSA.

### *Computed Tomography*

**Fig. 7:**
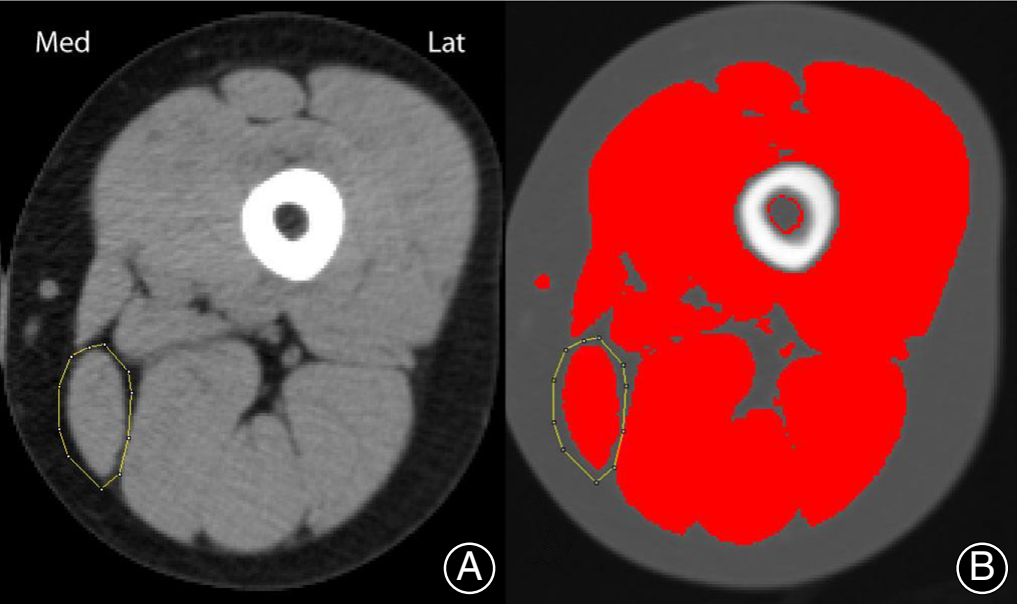
Computed tomography (CT) image of left thigh (A) and that treated with ImageJ (B)^78^.

Computed tomography scanning is an X‐ray radiological imaging technique that yields transverse tomographic images reflecting with high accuracy the spatial distribution of X‐ray attenuation in the part examined^109^ and gives an exact and accurate cross‐sectional picture of the soft tissues^110^. X‐ray attenuation is indicative of the type of tissue under scan and can, therefore, be used to construct an image of the internal structure of the body^77^. Because of the high‐density resolution and collimation system of CT, it can distinguish small differences in soft tissues without the interference of the extra‐lamellar structure. CT is mainly used for CSA measurement of thigh muscle^75^, ^78^, ^79^, ^80^. The CT image of the left thigh for CSA measurement is shown in Fig. 7.

With the rapid development of the CT scanning technique, fast image acquisition and high spatial resolution images were realized. The use of new medical imaging software made it possible to measure areas within specified attenuation limits. Peripheral quantitative CT (pQCT) is commonly used for soft tissue area quantification by segmenting regions representing different tissues. Sherk compared human muscle and fat CSA measurements between MRI scans and pQCT and found that CSA did not differ significantly between MRI and strongly filtered pQCT images^79^. Haggmark's research shows that CT is an accurate way of measuring the size of different muscle bellies^75^. Clement *et al*. demonstrated that reliable and accurate measurements of the articular surfaces can be measured using a freeform tool on CT scans^111^. Diffusible iodine‐based contrast‐enhanced CT (diceCT) and μCT, which includes both iodine‐based staining and the use of other staining agents, was developed recently as a means of visualizing muscle portions *in situ*, which enabled high‐resolution data to be collected^103^.

Computed tomography and new medical imaging software enable easy and rapid assessment of the CSA of soft tissues. These are commonly used diagnostic techniques and can provide an exact and accurate cross‐sectional picture of the soft tissues. CT scans provide much clearer information than plain X‐rays^112^ and a CT scan is also expected to be more accurate than ultrasound scanning because it can clearly define the boundary at the interface between fat and muscle^113^. However, it is quite a complex and expensive technique that is not adaptable to all kinds of tests.

## Molding Techniques

**Fig. 8:**
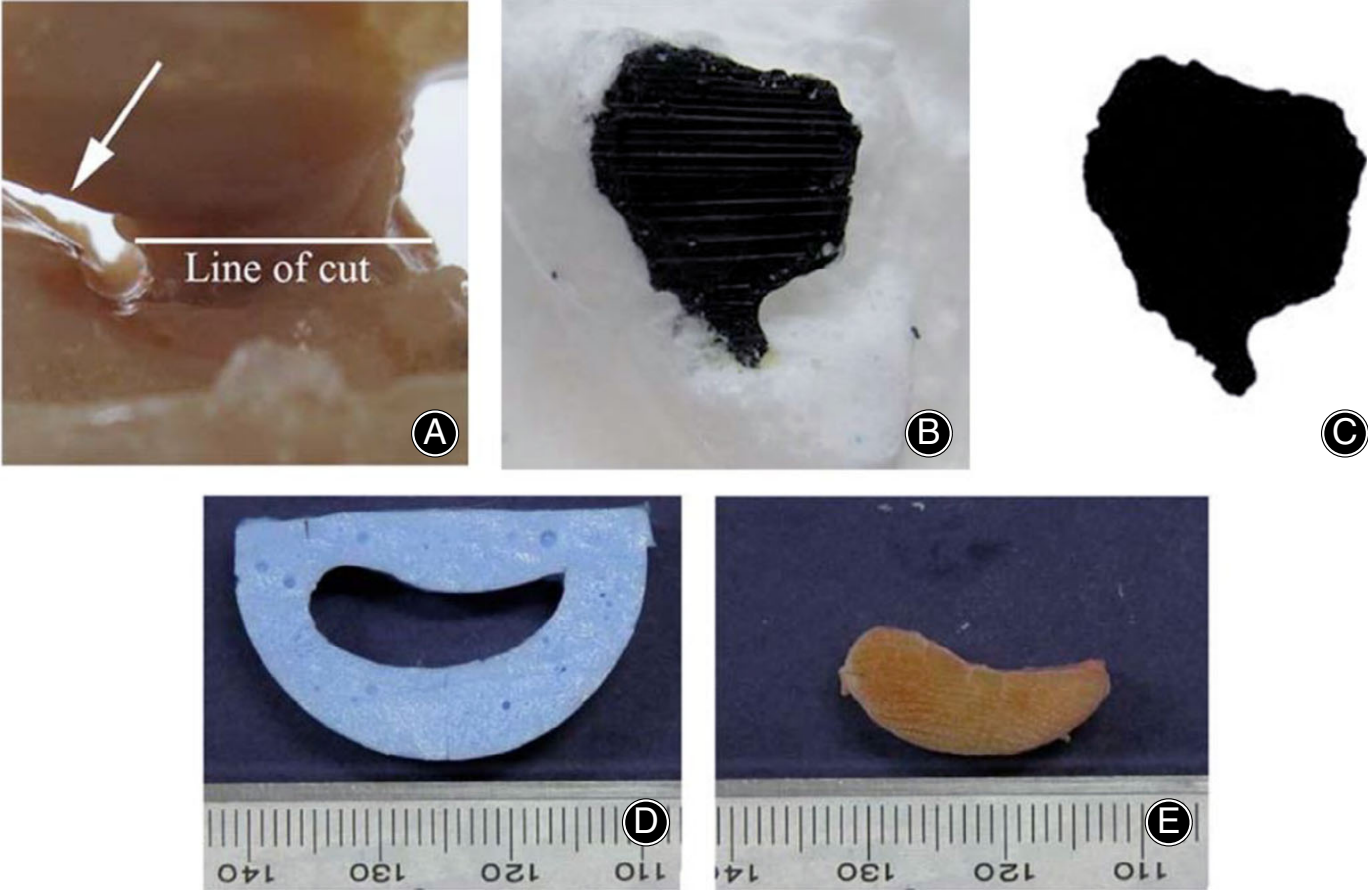
Silicone rubber/polymethylmethacrylate (PMMA) molding technique measuring the cross‐sectional area (CSA) of foot ligament: (A) interosseous 4th metatarsal‐5th metatarsal (IM4M5) bone‐ligament‐bone specimen; (B) silicon rubber mold of IM4M5 showing cross‐section of ligament; and (C) PMMA casting of IM4M5 specimen^27^. Alginate molding technique measuring equine CSA of equine superficial digital flexor tendon (SDFT): (D) transverse section through the alginate mold of SDFT (E) a photograph of a transverse section through the tendon used to make the mold at the same level^36^.

Molding techniques measure the CSA of soft tissues by making a cast of soft tissues and measuring their cast directly. In this way, accurate CSA and morphological information including concavities of soft tissue can be accessed with no damage to the soft tissue. According to molding materials, molding techniques can be divided into the silicone rubber/PMMA molding technique^26^, ^27^, ^81^, ^82^ and the alginate molding technique^36^, ^37^, ^83^, ^84^, ^85^, ^106^, examples of which are shown in Fig. 8.

The silicone rubber/PMMA molding technique was developed by Race and Amis in 1996. They determined the CSA of soft tissue by making a silicone rubber cast and a PMMA replica of a specimen^26^. Then the PMMA replica was sliced and stained, and the CSA was calculated by square counting. A curing agent was mixed with liquid silicone rubber to speed up the curing process. However, due to the PMMA shrinkage and square counting method, there was a systematic underestimation of 6.2% and a random error of 1.8% after correction. The problem of PMMA shrinkage was solved by optimizing molding materials using a formula by Schmidt *et al*.^27^. The application of camera and Image analysis software greatly improved the measurement accuracy, to 2%.

The silicone rubber/PMMA molding technique is accurate, non‐destructive, allows measurement of non‐uniform shape specimens, and can obtain morphological information. There are still a few potential limitations. It is time‐consuming and soft tissue needs to be exposed to air for 2 h while molding, which may cause dehydration. If moisturizing tissue with saline, excess saline could gather and create bubbles in the mold and, thus, change the CSA. All of this reduces the success rate of casting.

The alginate molding technique uses dental alginate impression materials as molding materials^36^. After the molding process, alginate mold was sliced and photographed. Results show that the alginate molding technique has high measurement accuracy (0.8%) and a short operation time (approximately 5 min). The alginate mold is easy to cut so a thin blade can be used to obtain a flat cut and the shrinkage is almost 0% within 10 min. Casting is not needed. It can accurately measure an area of 3 mm^2^, which is smaller than for the silicone rubber molding method (7 mm^2^). However, this technique cannot be applied to measuring the CSA of more complex soft tissue (such as the anterior cruciate ligament) or soft tissue with bony ends that are hard to pull out of the mold.

## Laser Techniques

### *Laser Micrometers*

Laser micrometers can obtain the cross‐sectional shape and area of soft tissues by reconstructing the cross‐sectional shape based on the width measurements. The widths of specimens are obtained using collimated laser beams.

**Fig. 9:**
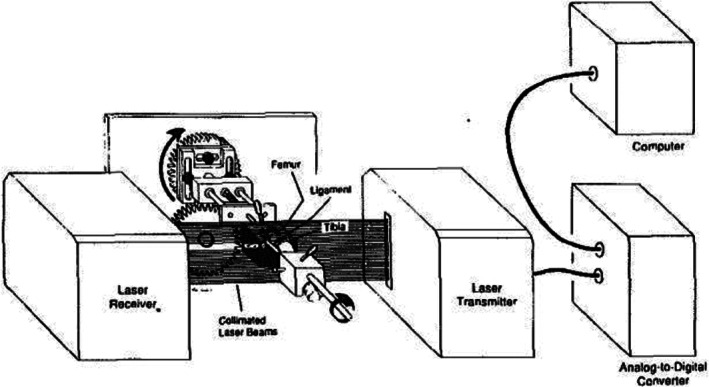
Schematic drawing of laser micrometer system and the clamp assembly. The specimen profile widths are measured by a laser telemetric system, which is a microprocessor‐controlled device that sends collimated scanning laser beams from the transmitter to the receiver. When an object is placed within the laser beams, a precise shadow is cast onto the receiver. This information is then transmitted to a controller where the width of the object is reported. The specimen's profile width (PW) and a reference distance (RD, from the top of the profile to the upper edge of the laser beams) are measured at each angular increment as the specimen is rotated through 180°^39^.

**Fig. 10:**
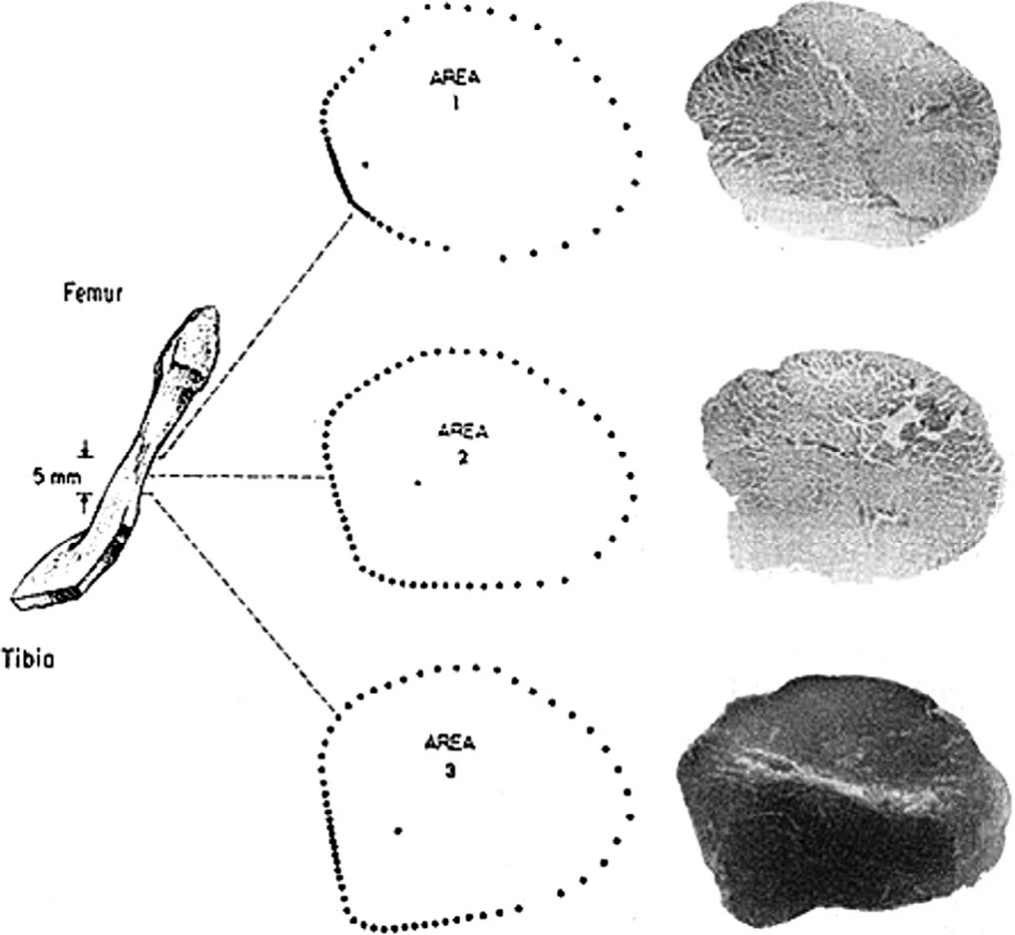
The reconstructed cross‐sectional shapes of porcine anterior cruciate ligament (ACL) obtained using laser micrometer (middle) are shown together with the corresponding histological sections (right).^38^

In 1988, Lee and Woo developed a non‐contact CSA measurement technology based on laser micrometers (shown in Figs 9 and 10)^38^. During width measurement, specimens were placed perpendicular to collimated laser beams and rotated 180°. Then, the specimen's cross‐sectional shape was reconstructed based on width measurements following a specially designed algorithm, and the CSA was calculated using Simpson's rule. This study compared the CSA of eight porcine ACL specimens measured by laser micrometers and the area micrometer method, and revealed that the results obtained by area micrometer method was 17% lower than those obtained by laser micrometers. In 1990, Woo made the acquisition process for the laser micrometer system automated and fully computerized, which sped up the process. He applied this method to determine the cross‐sectional shape of a rabbit medial collateral ligament and ACL and the results showed that the measurement accuracy of laser micrometer was less than 2%^39^, ^114^. In Harner's study, specimens are placed perpendicular to the ground, which can decrease the deformation of specimens during rotation^115^. Race and Amis found, compared to the replica method, that the laser micrometer method overestimate CSA by an average of 2.3 and 1.5% (SD) for tendons^26^. In 2010, Bruneau developed an optic micrometer that submerges the tendon into a measuring compartment filled with cold saline solution and estimated the CSA of rat tail tendons within a 2% margin of error^97^.

Laser micrometer is non‐destructive, can obtain the geometric information, and allows multiple measurement. However, the laser micrometer is affected by specimen geometry. The concave region is assumed to be flat during the image reconstruction and, thus, overestimated the CSA by 19% compared to the casting method^26^. Moreover, it needs all‐round visibility and, therefore, the removal of adjacent bones is required, which has negative impacts on implantation. The data acquisition process requires approximately 1–2 min for each cross‐section to collect numerous data points. There are also systematic errors associated with increments, and the operations are complicated and expensive.

### *Laser Reflection System*

The Laser Reflection System (LRS) measures the CSA of soft tissues by rotating the laser sensor around the soft tissue samples in a circular path. The cross‐sectional shape is reconstructed and the CSA is determined by the inner radius (r) of the specimen relative to the rotating center and the corresponding angle in polar coordinates using Simpson's rule.

In 1995, Chan developed a laser reflectance system using position‐sensing detector (PSD) laser displacement sensors^116^. PSD are more sensitive to surface properties (i.e. hydration and opacity) than charge‐coupled device (CCD) laser displacement sensors. With the development of CCD, in 2006, Moon developed and validated a CCD laser reflectance system composed of a CCD laser sensor and rotary motion table (shown in Fig. 12)^90^ and found that the accuracy of the system was less than or equal to 2.0% with a repeatability of 0.0%. The cross‐sectional shapes obtained with this system were in good agreement with those obtained by laser micrometer and the laser micrometer system overestimated CSA by approximately 6%. Favata improved the CSA measurement method for small connective tissues by using a laser triangulation sensor in combination with two LVDT to acquire the thickness and x and y displacements^91^. This technique has been used in animal^92^, ^93^ and human tendons^94^, ^117^. Pokhai developed a new laser reflectance system capable of measuring changing CSA of soft tissues during tensile testing (shown in Fig. 13), which was designed to be installed on an Instron 8872 servohydraulic test machine; the measurement accuracy of this system was less than 4.3%^40^. It has been applied to the CSA measurement of rat tendons^93^ and human forearm tendons^94^.

**Fig. 11:**
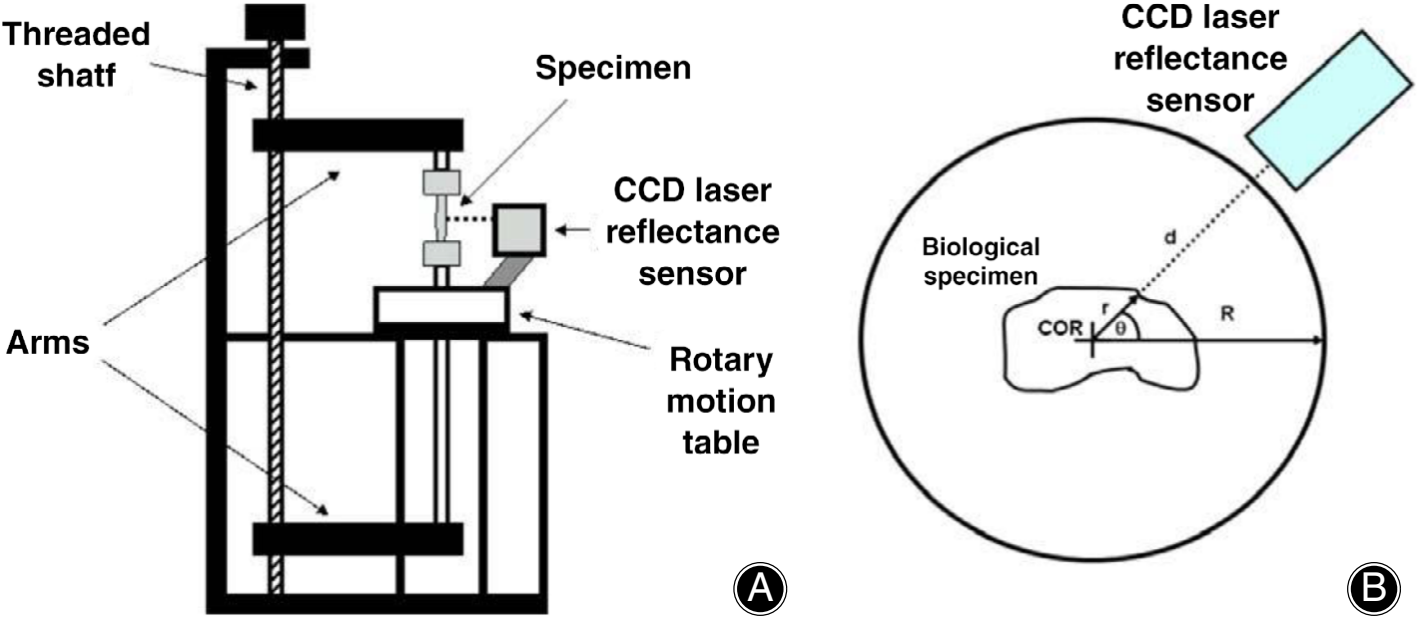
(A) The schematic of charge‐coupled device (CCD) laser reflectance system, in which a CCD laser displacement sensor was mounted onto a rotary motion table. The frame has a threaded shaft connected to two arms that hold the specimen and allows for vertical translation relative to the CCD laser reflectance system. (B) A diagram depicting an overhead view of the CCD laser reflectance system with a biological specimen over the center of rotation (COR) of the system. “R” represents the total radius of the system, “d” represents the distance to the surface of the specimen, and “r” represents the inner radius of the specimen with its respective angle (y)^90^.

In conclusion, the LRS can successfully measure the CSA of soft tissues with concavities in an accurate, repeatable, and rapid manner (shown in Fig. 11). It is a non‐contact, non‐destructive, and accurate tool for CSA measurement and is the first device that could measure changing CSA during tensile testing. However, it is too slow to use during mechanical testing because a complete revolution of the LRS takes over 20 s to complete and the strain rate must be slow (approximately 2 mm/min), which may introduce error caused by the viscoelastic of soft tissues^40^. The rotation center should be in the cross‐section of the specimen. Another limitation of the CCD laser sensor is that it does not perform as well for semitransparent surfaces, so the tissues need to be stained. The accuracy was affected by specimen size as well as the spot beam diameter. Therefore, only specimens larger than 20 mm^2^ were considered currently^90^. Moreover, when the angle between laser beams and the target surface is steep, the artifact of the shape of the cross‐section is generated due to the light reflecting far from the charge‐coupled device CCD sensor^95^.

**Fig. 12:**
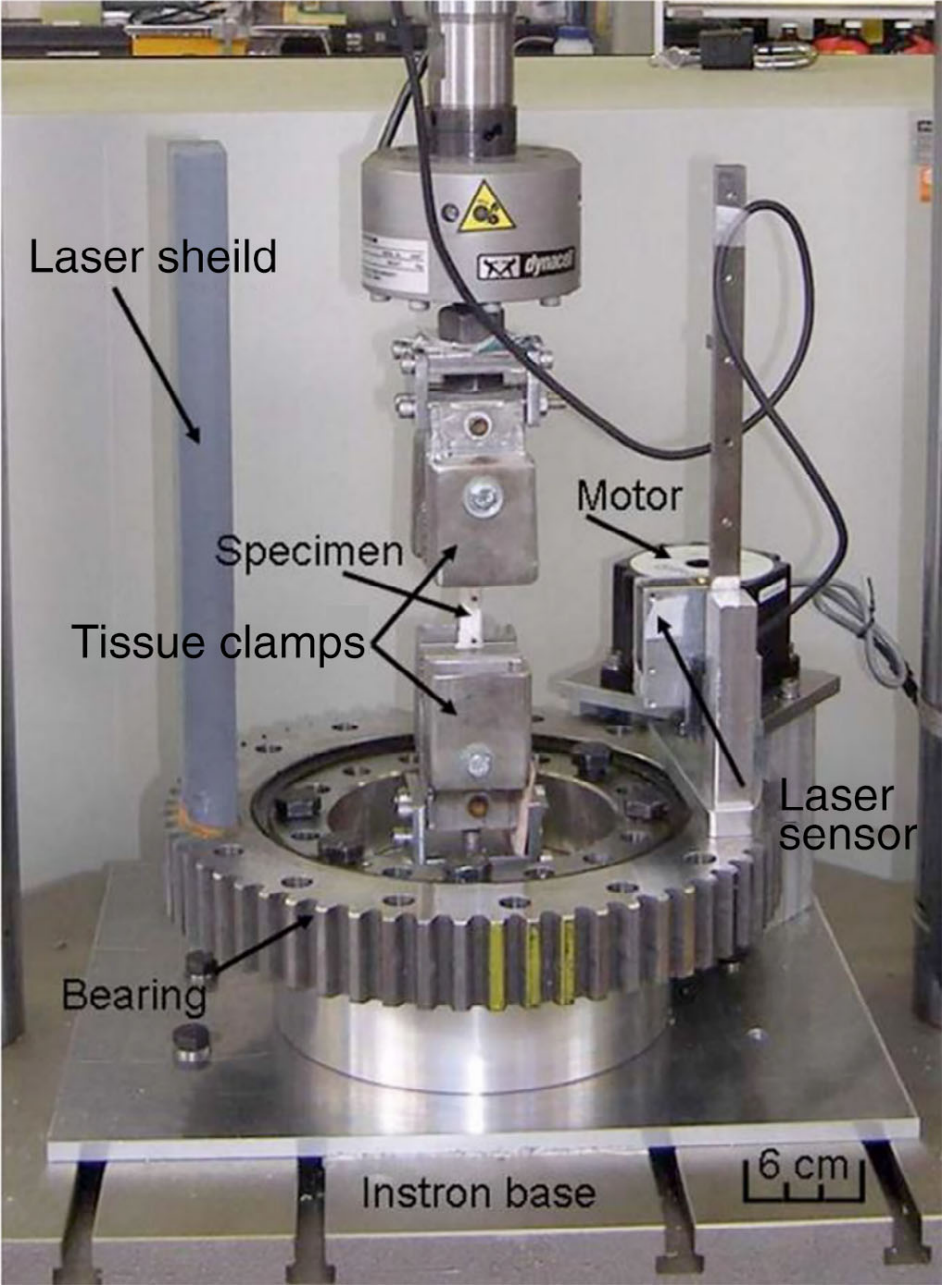
Photograph of the laser reflectance system installed on an Instron 8872 servohydraulic testing machine to allow cross‐sectional area measurement during tensile Testing^40^.

**Fig. 13:**
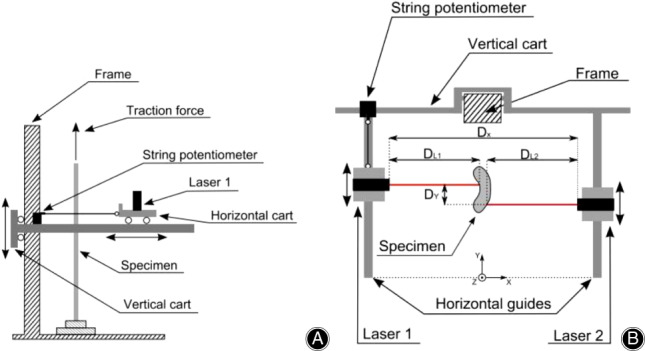
(A) Lateral view and (B) top view of linear laser scanner. DX and DY are the distance between lasers and the offset imposed to avoid mutual interference. DL1 and DL2 are the distances measured by each laser between itself and the specimen surface^95^.

**Fig. 14:**
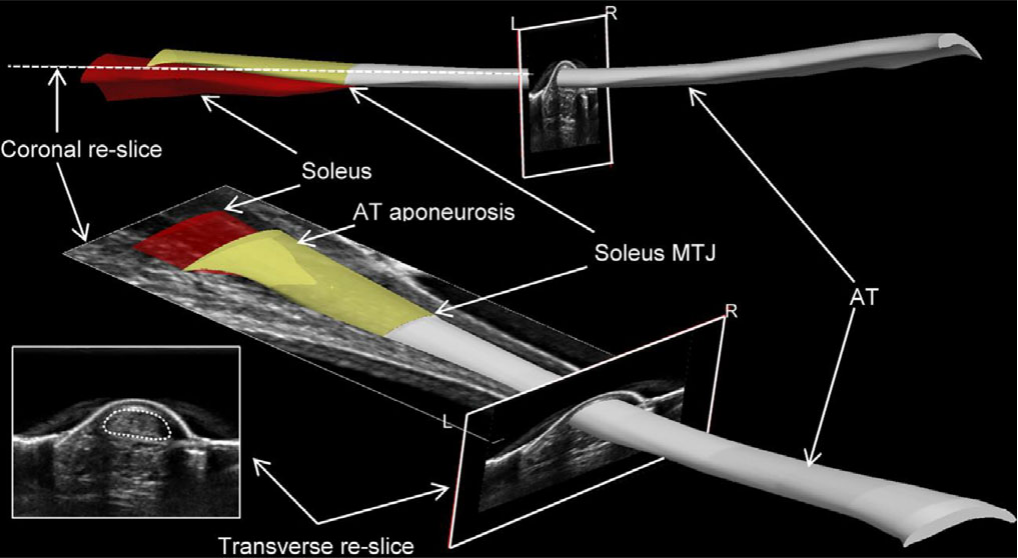
Freehand three‐dimensional ultrasound (FUS) reconstruction of the Achilles tendon (AT). Three‐dimensional ultrasound image reconstructions were created in Stradwin using manually digitized cross‐sections (dotted white lines within inset) of the tendon segmented at approximately 10‐mm to 15‐mm intervals along the length of the tendon^101^.

### *Linear Laser Scanner*

The linear laser scanner (LLS) is composed of two CCD laser reflectance devices mounted facing each other on two carts sliding on horizontal and parallel linear guides, with the specimen placed vertically between the two horizontal guides, the schematic of which is shown in Fig. 13 ^95^. During measurement, the lasers are moved along the guides to sweep the specimen in the x−y plane and measure the distance from the laser to the specimen surface. Then image reconstruction is processed to obtain a cross‐sectional shape and the CSA is calculated using MATLAB.

In 2010, Vergari *et al*. designed an LLS which could obtain accurate and repeatable CSA (less than 2% error) with short acquisition time (within 2 s per measurement) and, thus, could perform CSA measurements under continuous tension^95^. The LLS has been used to measure the CSA of equine superficial digital flexor tendons^96^.

The LLS Linear Laser Scanner is accurate, repeatable, and fast and easy to assemble and operate. It can adapt to various types of testing machines and is capable of moving to follow a defined zone on the specimen during testing. The system does not need precise centering of the sample and can perform noncontact measures during mechanical testing. However, this device cannot precisely acquire steep concavities due to the reflection of the laser beams. The specimen maximum measurable thickness (45 mm) is also a limitation.

## Three‐Dimensional Scanning Techniques

Three‐dimensional scanning techniques access the CSA by sectioning the 3D model of soft tissues acquired by optical, laser, or ultrasound techniques, such as SLS and 3D freehand ultrasound.

The optical 3D scan system was developed by Hashemi *et al*. using a commercially available photographic scanner, 3D Scantop (Olympus America), to construct the 3D image of a human ACL^98^. This system is accurate, easy to apply, and affordable, at US$5000. It also allows the determination of CSA at any position of the tissues and all relevant information can be extracted from one single application of the method. However, as with most optical techniques, the system cannot detect surface concavities.

Freehand 3D ultrasound (FUS) combines brightness mode (B‐mode) USI with a motion analysis system to generate 3D reconstructions of anatomic structures *in vivo* ^100^, ^101^, ^118^.

Reconstructed 3D volume is created using a rigid body calibration method to transform sequential 2D images into a global coordinate system, which is subsequently used for object segmentation. Three‐dimensional ultrasound has been validated to have excellent repeatability^99^ and can provide reliable measurements^101^. However, accurate 3D reconstructions can only be achieved when ultrasound images are obtained during static conditions and the accuracy of results may not be representative of the accuracy of measurements *in vivo* ^101^. Figure 14 shows the application of FUS on Achilles tendon CSA measurement.

Structured light scanning can reconstruct the 3D digital models of soft tissue using a variation on stereophotogrammetry. Hayes *et al*. developed a CSA measurement technique using a commercially available structured light scanner named Artec Spider and integrating it with a custom mechanical rig permitting 360° acquisition of the morphology of soft tissues^102^. The reconstructed 3D model was then used to measure the CSA of the tendon. Specimens should be light coated with flour to reduce excessive reflection. This technique can measure the entire shape without contact but long‐term reliability and the cost of the device may be potential limitations.

Other techniques, such as the 3‐D Scantop imaging system^98^, the 3 T scanner (Signa PET/MR,GE Healthcare) ^104^, the three‐dimensional CT^112^ (3DCT: three‐dimensional gray‐scale‐images) that may be viewed from different angles and in real‐time rotation), can also be used to obtain 3D images and compute the CSA.
